# Microcystic Adnexal Carcinoma of the Head and Neck: Diagnostic Work-Up and Contemporary Treatment Strategies—A Narrative Review

**DOI:** 10.3390/cancers18203273

**Published:** 2026-10-11

**Authors:** Lawik Revend, Florian Dudde

**Affiliations:** 1 Department of Orthopedic and Trauma Surgery, Division of Plastic Surgery, Army Hospital Berlin, 10115 Berlin, Germany; 2 Department of Oral and Maxillofacial Surgery, Army Hospital Hamburg, Lesserstraße 180, 22049 Hamburg, Germany

**Keywords:** microcystic adnexal carcinoma, head and neck, Mohs micrographic surgery, perineural invasion

## Abstract

**Background:** Microcystic adnexal carcinoma (MAC) is a rare cutaneous adnexal malignancy characterized by slow clinical growth but pronounced local aggressiveness, extensive subclinical infiltration, and a propensity for perineural invasion. Owing to its rarity and often inconspicuous clinical presentation, diagnosis and treatment remain challenging, particularly in the anatomically complex head and neck region. **Methods:** A narrative review of the literature was performed using PubMed/MEDLINE and Embase, covering publications available up to August 2026. Relevant studies addressing the clinical presentation, diagnostic work-up, histopathology, imaging, surgical and non-surgical treatment, recurrence, and follow-up of MAC were reviewed, with particular emphasis on MAC arising in the head and neck region. **Results:** Adequate deep biopsy extending into the subcutaneous tissue is essential for reliable diagnosis, as superficial sampling may result in misdiagnosis. Contrast-enhanced magnetic resonance imaging may be considered when deep infiltration or clinically significant perineural spread is suspected, although MAC-specific imaging evidence is limited. Complete surgical excision with comprehensive microscopic margin assessment represents the cornerstone of treatment. Mohs micrographic surgery or staged excision with comprehensive peripheral and deep margin assessment may be favored when technically feasible; comparative evidence is retrospective and does not establish a survival advantage. More extensive oncological resection may be required for tumors involving deep structures or major nerves. Elective treatment of the clinically negative neck is generally not indicated because regional metastases are uncommon. Radiotherapy has a selective role in patients with high-risk features, unresectable disease, or inadequate surgical margins, but supporting evidence remains predominantly retrospective. **Conclusions:** Management of head and neck MAC should focus on accurate diagnosis and durable local and perineural disease control. Available evidence and expert guidance favor margin-controlled surgery, with radiotherapy considered selectively; the optimal treatment and surveillance strategies have not been prospectively established. Given the potential for late recurrence, prolonged clinical surveillance is warranted.

## Simple Summary

Microcystic adnexal carcinoma is a rare skin cancer that occurs mainly on the head and neck. Although it may look small and grow slowly, it can extend deeply beneath the skin and sometimes along nerves. This review summarizes the available evidence on diagnosis and treatment. A sufficiently deep tissue sample is important because a superficial biopsy can miss the characteristic features. Imaging may help when deeper spread is suspected, but it cannot replace examination of surgical margins under the microscope. Surgery that carefully checks the edges and base of the removed tissue is generally favored when feasible. Radiotherapy may be considered for selected patients, and long-term follow-up is important because recurrence can occur years later. Most recommendations rely on small retrospective studies and expert guidance, so treatment should be adapted to the individual patient.

## 1. Introduction

Microcystic adnexal carcinoma (MAC) is a rare malignant cutaneous adnexal neoplasm characterized by slow clinical growth but pronounced local aggressiveness [1,2]. Sclerosing sweat duct carcinoma is a historical synonym; syringomatous carcinoma has also been used in the literature [2]. MAC shows follicular and sweat duct differentiation, but its precise cell of origin remains unresolved. A pluripotent adnexal epithelial precursor is a proposed explanation rather than an established cell of origin [2,3]. Since its initial description in the early 1980s, MAC has emerged as a distinct diagnostic and therapeutic challenge because of its deceptively benign clinical appearance, deeply infiltrative growth pattern, frequent perineural invasion, and tendency to extend substantially beyond clinically apparent tumor margins [1,2,4,5]. Although distant and regional lymphatic metastases are uncommon, inadequate local treatment may result in repeated recurrence, extensive tissue destruction, neurological morbidity, and challenging reconstructive defects [6,7].

MAC demonstrates a marked predilection for the head and neck, particularly the central face [1,2,4,5,6,7]. Consequently, treatment frequently involves anatomically and functionally sensitive structures, including the lips, nose, periorbital region, cheek, scalp, and external ear. The discrepancy between limited surface involvement and extensive microscopic spread represents one of the defining characteristics of MAC and has important implications for biopsy technique, preoperative imaging, surgical planning, and margin assessment [8]. Because of the rarity of the disease, high-level prospective evidence is unavailable, and current recommendations are predominantly derived from retrospective series, systematic reviews, and expert consensus [1,2,4,5,6,7,8,9,10]. These sources support a practical clinical framework rather than a prospectively validated management algorithm [9,10,11,12]. This review summarizes current concepts regarding the diagnosis and treatment of MAC, with particular emphasis on tumors arising in the head and neck. Figure 1 summarizes the proposed approach and its principal evidence limitations.

## 2. Materials and Methods

This narrative review was conducted to summarize the current evidence regarding the diagnosis and management of MAC, with particular emphasis on tumors arising in the head and neck region. A literature search was performed using PubMed/MEDLINE and Embase, covering publications available up to August 2026. Search terms included combinations of “microcystic adnexal carcinoma”, “sclerosing sweat duct carcinoma”, “head and neck”, “Mohs micrographic surgery”, “wide local excision”, “perineural invasion”, “radiotherapy”, “imaging”, “recurrence”, and “metastasis”. Peer-reviewed English-language publications addressing the clinical presentation, diagnostic work-up, histopathology, imaging, surgical management, radiotherapy, systemic treatment, recurrence, and follow-up of MAC were considered. Priority was given to clinical practice guidelines, systematic reviews, larger retrospective studies, and case series. Owing to the rarity of MAC and the limited availability of higher-level evidence, selected case reports were additionally included when they provided clinically relevant information regarding uncommon presentations, advanced or metastatic disease, imaging findings, or specific therapeutic approaches. Reference lists of relevant publications were screened to identify additional pertinent studies. Selection was guided by relevance to the diagnostic or therapeutic question and the clinical information contributed, rather than by a predefined systematic eligibility protocol. Evidence from other skin or adnexal cancers was considered only for explicitly identified contextual comparisons.

Both authors independently reviewed the available literature. Duplicate references were subsequently removed, and the remaining publications were jointly discussed and assessed for their relevance to the review questions. The findings were interpreted and synthesized jointly within the framework of this narrative review, with an emphasis on clinically applicable diagnostic and therapeutic principles. No formal systematic study-selection protocol, risk-of-bias assessment, or quantitative evidence synthesis was applied. Selection and publication bias are therefore possible, and the review should not be interpreted as an exhaustive systematic evidence assessment. During revision, targeted source checks addressed the reviewers’ questions on immunohistochemistry, paraffin-section margin control, excision margins and emerging imaging methods, including the suggested ultrasound study. This was not a repeat systematic database search.

## 3. Clinical Presentation and Biological Behavior

MAC typically affects middle-aged and older adults and most frequently arises on chronically sun-exposed skin of the head and neck [1,2,4,9,10]. In the systematic review informing the 2019 clinical practice guideline, 54.1% of 1968 patients were women and 45.9% were men, indicating a modest female predominance rather than a marked sex difference [9]. Clinically, it usually presents as a slowly enlarging, firm, poorly defined papule, plaque, or subcutaneous nodule. The overlying epidermis may appear normal or demonstrate subtle induration or discoloration. Because the tumor often progresses over several years and may initially be asymptomatic, diagnosis is frequently delayed [7,8,9,10,11]. The relatively inconspicuous surface appearance contrasts with the biological behavior of the tumor. MAC demonstrates extensive infiltration through the dermis and subcutaneous tissue and may invade skeletal muscle, cartilage, bone, or other adjacent structures in advanced cases [13]. Histological tumor dimensions may therefore considerably exceed the clinically estimated dimensions. This phenomenon is particularly relevant in the head and neck, where even relatively small lesions may infiltrate critical anatomical structures.

Perineural invasion (PNI) is another characteristic feature of MAC [9,10,14]. Reported frequencies vary substantially between series, partly because of differences in pathological assessment and case selection [1,2,4,5,7,8,9,10,12,13,14]. PNI may remain an incidental microscopic finding or, in more advanced disease, result in pain, paresthesia, numbness, weakness, or other cranial nerve deficits. Clinical neurological symptoms should therefore raise suspicion of extensive perineural tumor spread. Despite its locally aggressive behavior, MAC has a comparatively low metastatic potential [15]. Regional lymph-node and distant metastases have been described but remain exceptional [9,10,15,16]. Consequently, the principal therapeutic challenge is durable local control rather than prevention of systemic dissemination. Figure 2 illustrates possible subclinical and perineural extension; the pattern and extent vary between patients.

## 4. Clinical Examination

Careful examination should determine the clinically apparent extent of disease and identify potential signs of deep or perineural invasion. Palpation is particularly important because induration may extend considerably beyond the visible lesion. Neurological assessment should be incorporated into the head and neck examination [1,2,4,5]. Sensory abnormalities, pain, paresthesia, motor dysfunction, or cranial nerve deficits may indicate clinically significant perineural involvement and should prompt further imaging [9,10,14]. Previous scars and areas of recurrent disease should also be carefully evaluated because recurrent MAC may demonstrate particularly irregular and extensive subclinical spread. Routine examination of regional lymph nodes is appropriate, although clinically occult nodal metastasis is uncommon and elective treatment of a clinically negative neck is generally not indicated [9,10,15,16].

## 5. Imaging

Imaging should be individualized according to tumor size, anatomical location, recurrence status, clinical depth, and suspicion of involvement of nerves or adjacent structures [9,20]. Contrast-enhanced magnetic resonance imaging (MRI) may be considered when clinically significant perineural spread or deep extension is suspected [9,21]. MAC-specific evidence is largely case-based and does not establish diagnostic accuracy, routine indications, or an optimal imaging protocol [21]. Assessment of the relevant nerve pathway toward the skull base and complementary computed tomography (CT) for suspected bone involvement follow general head and neck oncological imaging principles; their diagnostic performance has not been validated specifically for MAC. Cross-sectional imaging may be useful in large, recurrent or deeply infiltrating tumors when knowledge of the relationship to muscle, orbit, major nerves or bone could affect surgical planning. Routine systemic staging with positron emission tomography/computed tomography (PET/CT) is generally not warranted in localized MAC because distant metastatic disease is rare [9,10,15,16]. PET/CT or additional systemic staging may instead be considered for unusually advanced disease, suspicious lymphadenopathy, or clinical findings suggestive of metastatic spread. Imaging cannot exclude microscopic extension or replace histological margin assessment.

High-frequency ultrasound (HFUS) is an emerging adjunct for assessing the depth and local extent of cutaneous tumors. Popescu et al. reported agreement between measurements obtained with a portable 20 MHz HFUS device and histopathological tumor depth in basal cell carcinoma (BCC), while also examining systematic measurement differences and a depth-correction model [22]. These are BCC data, not evidence validating HFUS in MAC. The findings provide a rationale for future MAC studies, but neither the correction model nor assumptions about diagnostic accuracy can currently be transferred to MAC. HFUS should not be presented as an established method for defining MAC surgical margins or excluding perineural disease.

## 6. Biopsy and Histopathological Diagnosis

Histopathological examination remains the foundation of MAC diagnosis. However, obtaining an adequate biopsy is particularly important because superficial sampling represents a major source of diagnostic error. Current evidence-based recommendations emphasize that a biopsy performed for suspected MAC should extend through the dermis and include subcutaneous tissue whenever anatomically feasible [1,9,10]. In a retrospective series of 48 patients, 13 lesions (27%) had been misdiagnosed on a prior biopsy, illustrating the diagnostic challenge [17]. Suitable approaches include a deep punch biopsy reaching the subcutis or an incisional biopsy containing the full dermis and subcutaneous tissue, selected according to lesion size and anatomical constraints. Excisional biopsy may be appropriate for a small lesion if it does not compromise subsequent definitive treatment. The critical requirement is sufficient depth and representative tissue; superficial shave biopsies and shallow punch samples may capture only the upper component of the lesion, where keratinizing structures and relatively bland epithelial nests can mimic benign adnexal tumors or well-differentiated keratinocytic lesions. Consequently, MAC may initially be misdiagnosed as basal cell carcinoma, squamous cell carcinoma, desmoplastic trichoepithelioma, syringoma, or another adnexal neoplasm.

Histologically, MAC typically demonstrates a poorly circumscribed infiltrative proliferation extending deeply into the reticular dermis and subcutaneous tissue [1,23]. The superficial component often contains keratin-filled microcysts and nests of relatively bland squamoid cells, while deeper areas demonstrate cords and duct-like structures embedded in a dense desmoplastic stroma [3,23]. Cytological atypia and mitotic activity may be surprisingly limited despite extensive infiltration [3,23]. Perineural invasion should be specifically assessed and documented.

Immunohistochemistry is an adjunct to morphology. Tumor cells express epithelial cytokeratins, including high-molecular-weight cytokeratins, while epithelial membrane antigen (EMA) and carcinoembryonic antigen (CEA) can highlight ductal differentiation [3,24]. CK7 may label ductal lumina, and CK15 and CK19 may contribute to the differential diagnosis, but their expression is variable across studies [24,25]. BerEP4 is often negative or focal in MAC, in contrast to the usually diffuse staining of morpheaform BCC, but positive MAC cases occur; neither BerEP4 nor a single cytokeratin establishes or excludes the diagnosis [24,25]. Immunophenotypic overlap with desmoplastic trichoepithelioma also limits specificity. Deep infiltrative architecture, subcutaneous extension and perineural involvement must be interpreted with the staining panel. Difficult cases should be reviewed by a dermatopathologist or head and neck pathologist familiar with adnexal tumors.

## 7. Surgical Treatment

Surgical removal with complete microscopic margin control is generally favored for resectable MAC on the basis of observational evidence and expert guidance [9,10,17,18,19,26,27,28]. The infiltrative growth pattern and substantial discrepancy between clinical and histological tumor dimensions make conventional excision based solely on predetermined margins problematic. Mohs micrographic surgery (MMS) may be favored when technically and anatomically feasible [9,17,18,19]. Its principal advantage is comprehensive assessment of peripheral and deep surgical margins while preserving uninvolved tissue [9,18,19,26]. This is particularly relevant in the head and neck, where preservation of functional and aesthetically important structures must be balanced against the requirement for complete tumor clearance. Another important advantage of MMS is its ability to reveal the true extent of subclinical disease [17,18,19]. In a 48-case series, the final defect surface area after complete MMS averaged approximately four times the clinically apparent lesion size [17]. Surgeons should therefore anticipate potentially substantial defects even when the initial lesion appears relatively limited. Current comparative data support improved initial margin control with MMS, whereas a clear overall survival advantage over WLE has not been demonstrated [18,28]. These non-randomized comparisons are susceptible to selection bias and differences in tumor complexity and follow-up.

Staged excision using formalin-fixed, paraffin-embedded sections (often termed “slow Mohs”) is another approach to comprehensive peripheral and deep margin assessment. It requires mapped, systematic examination of the margins, with further excision as indicated before definitive reconstruction; routine sampled permanent sections alone are not equivalent. In a retrospective nine-patient facial MAC series, Palamaras et al. described persistent tumor after frozen-section MMS in two periocular cases with PNI and proposed paraffin-section assessment for selected difficult cases [29]. This small series supports consideration of paraffin sections when frozen-section interpretation is uncertain, particularly in recurrent or perineural disease; it does not demonstrate that slow Mohs is superior for all MACs.

Frozen sections can be used within a properly performed MMS procedure, but tissue processing and the extent of margin sampling are separate issues. Selected intraoperative frozen samples during conventional excision do not provide the same assessment as comprehensive peripheral and deep margin control. Subtle tumor cords in a sclerotic background and perineural involvement can complicate interpretation. Paraffin sections, additional levels or specialist review may be appropriate if clearance remains uncertain [9,29].

Conventional MMS has limitations in complex tumors involving deep anatomical structures [18,19,26]. Assessment may become challenging when tumor extends into skeletal muscle, cartilage, periosteum, bone, orbit, or major nerves. In such cases, multidisciplinary oncological resection with complete circumferential peripheral and deep margin assessment may be more appropriate. Wide local excision (WLE) remains an alternative when MMS is unavailable or anatomical circumstances favor conventional oncological resection [9,18,19,28]. In a 27-year retrospective cohort, 21 of 35 WLE-treated tumors (60%) had positive margins after the first surgical attempt, whereas all 34 MMS-treated tumors had negative margins after the initial procedure [18].

For WLE, the 2026 German S1 guideline on sweat gland carcinoma lists a clinical safety margin of at least 2 cm as an alternative to microscopically controlled surgery for MAC, while acknowledging the uncertain evidence and higher recurrence risk with WLE [30]. This is an expert-consensus recommendation, not a prospectively validated clearance threshold. Such margins may be impractical in the head and neck and cannot reliably encompass every deep or asymmetric extension [9,17,19]. Therefore, no fixed peripheral distance should substitute for careful assessment of peripheral and deep margins. Reconstruction should ideally be delayed until clearance is established, particularly before complex flap reconstruction.

## 8. Management of Deep and Perineural Disease

Head and neck MAC with extensive deep infiltration may require resection of involved muscle, cartilage, periosteum, or bone. Treatment should be guided by oncological principles rather than by the relatively indolent clinical appearance of the primary lesion. Perineural involvement requires particular attention [9,10,14]. Microscopic PNI involving small unnamed nerves can often be managed through complete surgical clearance. In contrast, involvement of a named nerve, clinical neurological deficits, or radiologically evident perineural tumor spread represents a substantially higher-risk situation. Resection of involved nerve segments may be considered when technically feasible, balancing clearance against functional morbidity in multidisciplinary discussion. Tumor spread along major branches of the trigeminal or facial nerves may necessitate more extensive skull-base-oriented evaluation and multidisciplinary treatment involving head and neck surgeons, dermatologic surgeons, radiologists, radiation oncologists, and, when appropriate, neurosurgical or skull-base teams. The management of named-nerve and skull-base disease is informed substantially by broader head and neck oncological practice, rather than comparative MAC-specific studies.

## 9. Management of the Neck

Regional metastasis is uncommon in MAC [9,10]. Rare nodal metastases have nevertheless been reported [6,15,16]. Therefore, elective neck dissection or sentinel lymph-node biopsy is not routinely recommended for patients with a clinically node-negative neck [9]. Suspicious lymph nodes should undergo ultrasound evaluation and needle biopsy, and confirmed nodal metastasis should prompt multidisciplinary consideration of therapeutic regional treatment [9]. This distinction is clinically important: extensive local invasion or PNI should not automatically be interpreted as evidence of increased lymphatic metastatic potential. The dominant biological behavior of MAC remains local and perineural rather than lymphatic.

## 10. Radiotherapy

The role of radiotherapy in MAC is less clearly defined than that of surgery [9,10,12]. Historically, concerns were raised regarding potential radioresistance or more aggressive behavior following irradiation [31], but subsequent retrospective experience has not shown consistent evidence of treatment-induced aggressive transformation [32]. Current evidence supports a selective rather than routine role for radiotherapy [9,10]. Postoperative radiotherapy (PORT) may be considered in patients with high-risk features for local recurrence, particularly when complete surgical clearance cannot be confidently achieved. Potential indications include persistently positive or very close margins when further surgery is not feasible, extensive PNI, involvement of a major named nerve, recurrent disease, and deeply infiltrative tumors in anatomically constrained locations [9,10,12,32,33]. In a dedicated MAC series of adjuvant radiotherapy, 14 head and neck cases treated after local excision achieved a crude local control rate of 93% [32]. The median dose was 55 Gy, and the authors suggested doses of at least 50 Gy [32]; this does not establish a universally applicable MAC dose standard. The uncontrolled, selected cohort cannot establish the incremental benefit of PORT over surgery alone, and these data should not be interpreted as evidence for routine PORT after complete margin-controlled excision. Broader cutaneous adnexal carcinoma series provide additional support for selective radiotherapy but are not MAC-specific [33]. Radiotherapy planning should account for the infiltrative nature of MAC. When clinically significant perineural spread is present, inclusion of the involved nerve pathway toward the skull base may be considered. This planning principle is extrapolated from broader head and neck oncology and has not been prospectively tested in MAC. This must be balanced against toxicity to adjacent critical structures, particularly for tumors involving the central face, orbit, or skull base. Definitive radiotherapy can also be considered in patients who are medically inoperable or when surgery would result in unacceptable functional or cosmetic morbidity [9,10]. However, evidence for definitive radiotherapy is limited to small series and case reports, and surgery with margin control remains preferred whenever feasible [9,10,12,32]. There is currently no convincing evidence supporting routine adjuvant radiotherapy following complete margin-controlled excision of an otherwise low-risk primary MAC. Treatment decisions should therefore be individualized within a multidisciplinary tumor board for advanced or high-risk cases.

## 11. Systemic Therapy

Systemic treatment has no established role in localized MAC. Conventional chemotherapy has demonstrated little evidence of meaningful activity and is not recommended as part of routine treatment [9,10]. Because metastatic MAC is exceptionally rare, evidence regarding modern targeted therapy or immunotherapy is extremely limited. Isolated responses to PD-1 inhibition have been reported in other metastatic primary cutaneous adnexal carcinomas, but these data are not MAC-specific and should not be directly extrapolated. In exceptional unresectable or metastatic MAC, molecular characterization may be considered within a molecular tumor board or clinical trial after local treatment options have been exhausted [9,34]. At present, however, no systemic regimen can be considered a standard treatment for MAC.

## 12. Recurrent Disease

Local recurrence remains one of the major clinical concerns in MAC and may occur years after initial treatment [1,2,4,5,6,9,10,35]. Recurrences are particularly associated with incomplete initial excision and may be more difficult to manage because previous surgery alters tissue planes and obscures the clinical extent of disease [9,10,26,27]. Recurrent tumors should therefore undergo renewed histological assessment and careful evaluation for deep and perineural extension. MRI should be considered, particularly when neurological symptoms are present or previous pathology demonstrated PNI [9,21]. Whenever technically feasible, recurrent disease should again be treated by margin-controlled surgery. Postoperative radiotherapy may be considered in multiply recurrent disease, especially in the presence of PNI, close or positive margins, or involvement of critical structures [9,10].

## 13. Follow-Up

Long-term surveillance is essential because MAC may recur after prolonged disease-free intervals [1,9,10]. Expert recommendations informed by a systematic literature review suggest examination every 6–12 months during at least the first five years following treatment [9]. This schedule has not been prospectively validated in MAC. Given the possibility of late recurrence, longer clinical surveillance is reasonable, particularly after treatment of recurrent, deeply infiltrating, or perineural disease [14]. Follow-up should focus on inspection and palpation of the surgical site, neurological assessment when relevant, and examination of regional lymph nodes [1,9,10,14]. Routine surveillance imaging is not supported by current evidence in asymptomatic patients after complete margin-controlled excision. In patients with extensive PNI or other high-risk characteristics, MRI may be considered according to symptoms and the initial pattern of disease; no MAC-specific imaging interval has been established. Patients should additionally be educated regarding self-examination and instructed to report new induration, nodularity, pain, numbness, paresthesia, or weakness in the treated region.

## 14. Conclusions

Microcystic adnexal carcinoma is a rare but clinically important malignancy of the head and neck whose indolent appearance contrasts with its capacity for extensive local and perineural invasion. Diagnostic delay and inadequate initial treatment remain major causes of morbidity. A deep biopsy incorporating the subcutaneous tissue is essential for accurate histopathological diagnosis, while contrast-enhanced MRI may support evaluation when deep or clinically significant perineural extension is suspected, although MAC-specific imaging evidence remains limited. Complete surgical excision with comprehensive microscopic margin assessment represents the cornerstone of treatment. MMS or staged surgery with comprehensive peripheral and deep margin assessment may be favored when feasible, although the comparative evidence is observational. More extensive oncological resection may be required when disease involves deep structures or major nerves. Radiotherapy may serve as an adjunct in selected high-risk cases and as an alternative for patients in whom adequate surgical treatment is not feasible, but the supporting evidence remains predominantly retrospective. The rarity of nodal and distant metastasis means that treatment should primarily focus on achieving durable local and perineural control rather than routine regional or systemic therapy. Because late recurrences can occur, prolonged clinical surveillance is warranted. Future multicenter registries and collaborative studies are required to better define risk stratification, indications for adjuvant radiotherapy, management of major-nerve involvement, and therapeutic options for the exceptionally rare patient with unresectable or metastatic disease. Prospective studies should also compare preoperative estimates of tumor depth and subclinical extension from MRI and emerging methods such as HFUS with mapped histopathological findings. Evaluation should include feasibility, measurement agreement and whether imaging meaningfully changes surgical planning; BCC-derived ultrasound models require MAC-specific validation before clinical adoption.

## Figures and Tables

**Figure 1:**
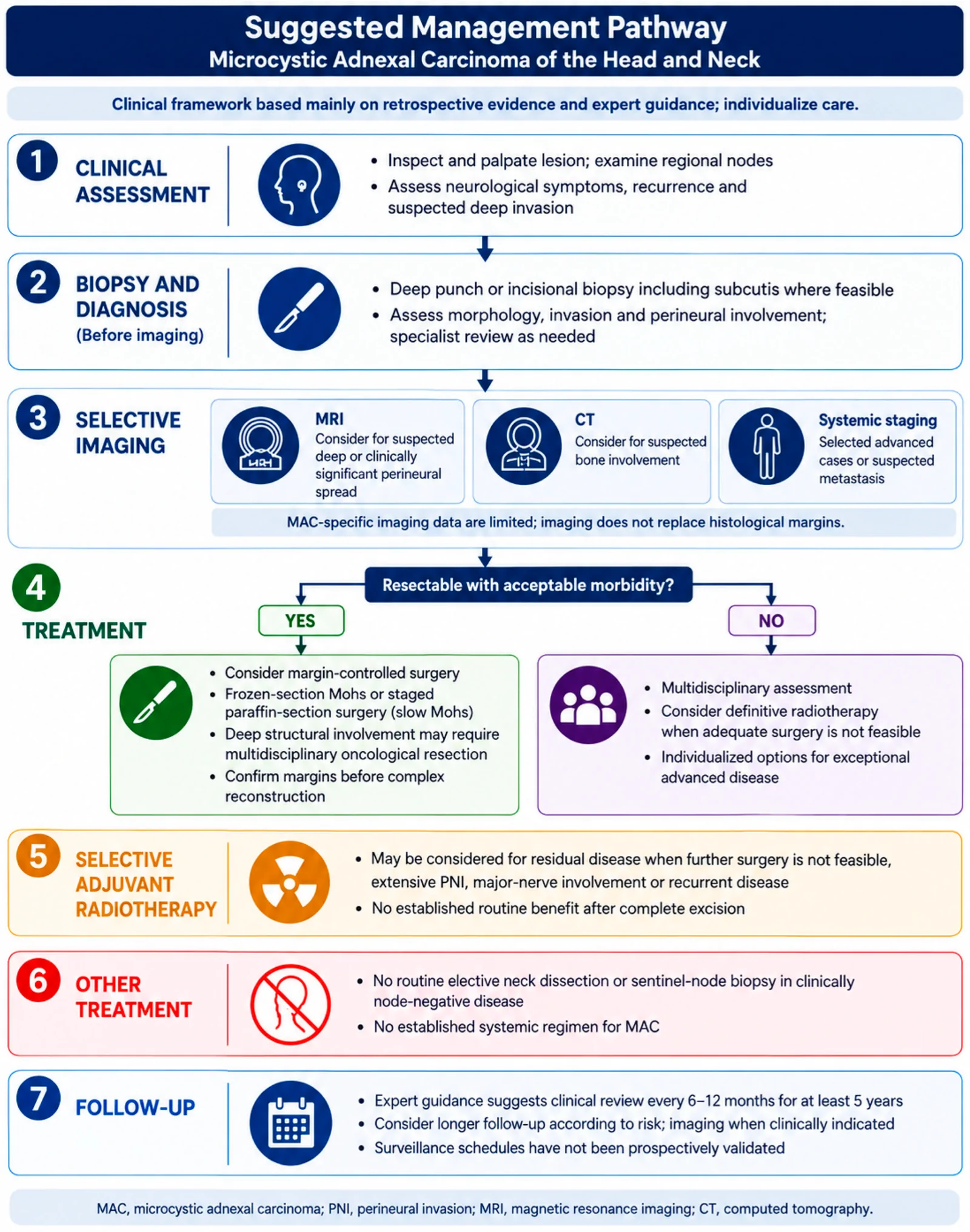
Suggested management pathway for head and neck MAC. This original schematic summarizes mainly retrospective evidence and expert guidance [9,10]. It is a clinical framework, not a validated decision rule; diagnostic sequencing and treatment should be individualized. Imaging is selective, radiotherapy is considered in selected situations, and surveillance intervals have not been prospectively validated. Both frozen-section Mohs surgery and staged paraffin-section margin control are shown as options. Abbreviations: CT, computed tomography; MAC, microcystic adnexal carcinoma; MRI, magnetic resonance imaging; PNI, perineural invasion.

**Figure 2:**
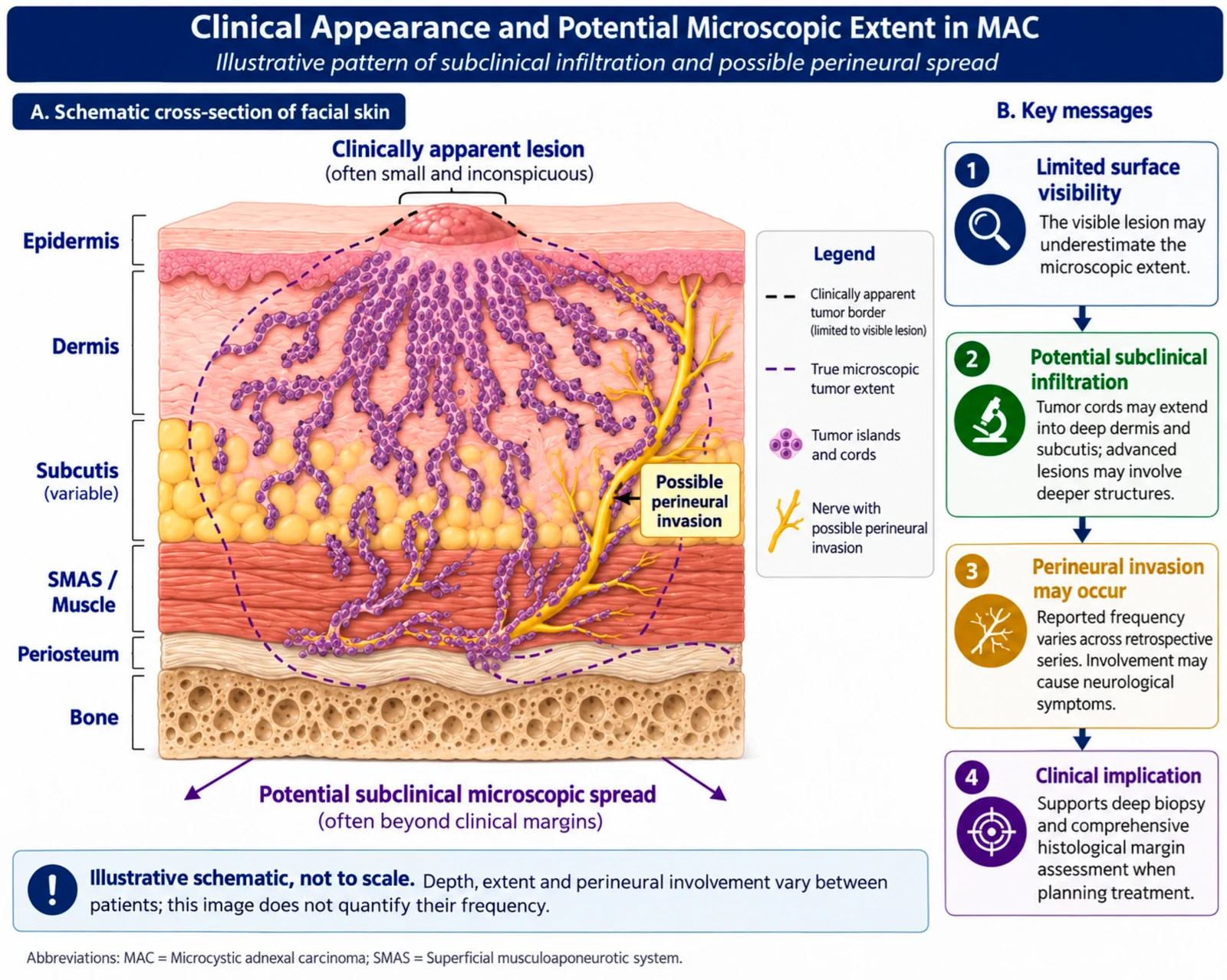
Clinical appearance and potential microscopic extent in MAC. Original illustrative schematic showing how the visible lesion may underestimate microscopic extension [17,18,19]. Perineural involvement is depicted as a possible finding, not as a universal feature or a quantitative estimate of its frequency. The drawing is not to scale and does not predict the depth or extent of an individual tumor. Abbreviations: MAC, microcystic adnexal carcinoma; SMAS, superficial musculoaponeurotic system.

## Data Availability

No new datasets were generated or analyzed in this narrative review. The sources supporting the discussion are cited in the reference list.
